# The Influence of Large Language Model-Generated Artificial Intelligence Risk Estimates on Clinical Decision-Making in Breast Imaging: A Simulation-Based Study

**DOI:** 10.3390/diagnostics16193241

**Published:** 2026-10-07

**Authors:** Ebru Dusunceli Atman, Sena Bozer Uludag, Çağlar Uzun, Gizem Agaran, Zeynep Eskalen, Kazim Burak Karaca

**Affiliations:** 1 Department of Radiology, School of Medicine, Ankara University, 06230 Altındag, Türkiye; senabozer@gmail.com (S.B.U.); cuzun77@yahoo.com (Ç.U.); gizemagaran@gmail.com (G.A.); zeynep_cotaoglu@hotmail.com (Z.E.); 2 Department of Radiology, Tokat State Hospital, Almus, 60950 Tokat, Türkiye; karacakazimburak@gmail.com

**Keywords:** BI-RADS, artificial intelligence, large language models, breast radiology education

## Abstract

**Background/Objectives:** Large language models (LLMs) are increasingly used as decision-support tools in radiology, yet their influence on clinical decision-making remains a significant concern. This study evaluates how artificial intelligence (AI)-generated malignancy risk estimates influence clinical decisions in breast imaging and assesses automation bias (AB) among radiology residents at different training levels. **Methods:** In this simulation-based study, 20 residents reviewed radiology reports for 15 synthetic breast imaging cases generated by ChatGPT-4 Turbo, each presented in either a concordant or discordant format. Participants were stratified into two groups based on their completion of a breast imaging rotation and evaluated each case by assigning a BI-RADS score, recommending management, and rating their confidence in the AI risk estimate. Diagnostic accuracy and independent predictors of AB were analyzed using Generalized Estimating Equations (GEE) to account for reader-level clustering. **Results:** Overall management accuracy was significantly lower in cases with discordant AI outputs (*p* = 0.008). A similar, albeit non-significant, decreasing trend was observed for BI-RADS accuracy. While advanced residents maintained high management accuracy regardless of AI concordance, early-stage residents showed a sharp decline in discordant cases (77.3% vs. 45.3%; *p* = 0.006). This decline was most critical in malignant cases for early-stage residents, where accuracy dropped from 92.0% to 48.0% (*p* < 0.001). In the multivariable GEE logistic regression model, training level was the only independent predictor of AB (OR: 0.09, 95% CI: 0.03–0.28, *p* < 0.001). **Conclusions:** In this simulation study, advanced clinical training was associated with lower susceptibility to AB in breast imaging, although training level alone is unlikely to be a sufficient safeguard. Safe integration of AI tools will require caution among junior trainees alongside system-level measures such as model transparency and institutional oversight.

## 1. Introduction

The integration of artificial intelligence (AI) into clinical radiology, alongside the development of large language models (LLMs), has created new opportunities by enabling advanced natural language processing to generate human-like textual outputs [1,2,3]. LLMs can generate clinically relevant text, including diagnostic reasoning, risk estimation, and report generation [4,5]. Similarly, machine learning models have demonstrated promising results in classification tasks using mammography, ultrasound (US), and magnetic resonance imaging (MRI) data [6,7,8,9,10]. Despite these advancements, the increased use of AI in clinical practice has led to a significant cognitive phenomenon known as “automation bias (AB)”—the tendency of operators to over-trust an automated system’s output [11,12,13]. Consequently, a physician may accept AI suggestions as accurate and reduce independent critical thinking, even when the system is incorrect. Furthermore, while AI systems are designed to augment human performance, erroneous outputs—often called “AI hallucinations”—can lead to significant diagnostic errors if uncritically followed.

In the specialized field of breast imaging, which relies on the Breast Imaging Reporting and Data System (BI-RADS) framework to minimize ambiguity in risk assessment and management, diagnostic precision is critical [14]. Despite this standardization, breast radiology remains one of the most cognitively demanding subspecialties, characterized by a significant learning curve for trainees. Residents must learn to distinguish malignant from benign lesions and to use clinical information to select appropriate management strategies.

The intersection between AI-generated decision support and trainee development is a subject of active research. Prior studies have shown that while AI can enhance the diagnostic performance of less experienced readers, this reliance may also introduce potential drawbacks [15]. A critical factor in mitigating AB is the level of specialized clinical training. Early-stage residents, who may not yet have the confidence developed through extensive practice, are theoretically more vulnerable to the misleading effects of discordant AI outputs, potentially leading to unnecessary biopsies or missed malignancies [16]. Notably, the current literature suggests that while more clinical experience reduces this vulnerability, it does not eliminate the risk of misdirection provided by AI [15,17].

Despite the growing body of literature on AI in radiology, there is a notable absence of research using LLM-generated synthetic scenarios to evaluate clinical decision-making. The primary experimental advantage of synthetic data is the ability to precisely control variables, enabling the direct identification of specific factors contributing to AB [18]. In this study, we evaluated how AI-generated malignancy risk estimates, derived from fully synthetic breast imaging scenarios generated by an LLM, influence the diagnostic performance of radiology residents. By involving residents at different training levels and employing both concordant and discordant AI outputs, this study explores the extent of AB and identifies factors that mitigate diagnostic over-reliance within a simulated clinical environment.

## 2. Materials and Methods

This simulation-based experimental study was designed to evaluate the influence of LLM-generated risk estimates on clinical decision-making in breast imaging. The prospective study protocol was approved by the Research Ethics Committee (Approval No: I11-998-25, dated 11 December 2025), and all participants provided informed consent prior to their inclusion in the study.

All participants were radiology residents with prior breast imaging exposure via educational modules or clinical rotations; for analysis, they were grouped into two cohorts based on their training level:

- Early-stage residents (R1–R3);
- Advanced residents (R4–R5), all of whom had completed a dedicated rotation in breast radiology.

**Figure 1:**
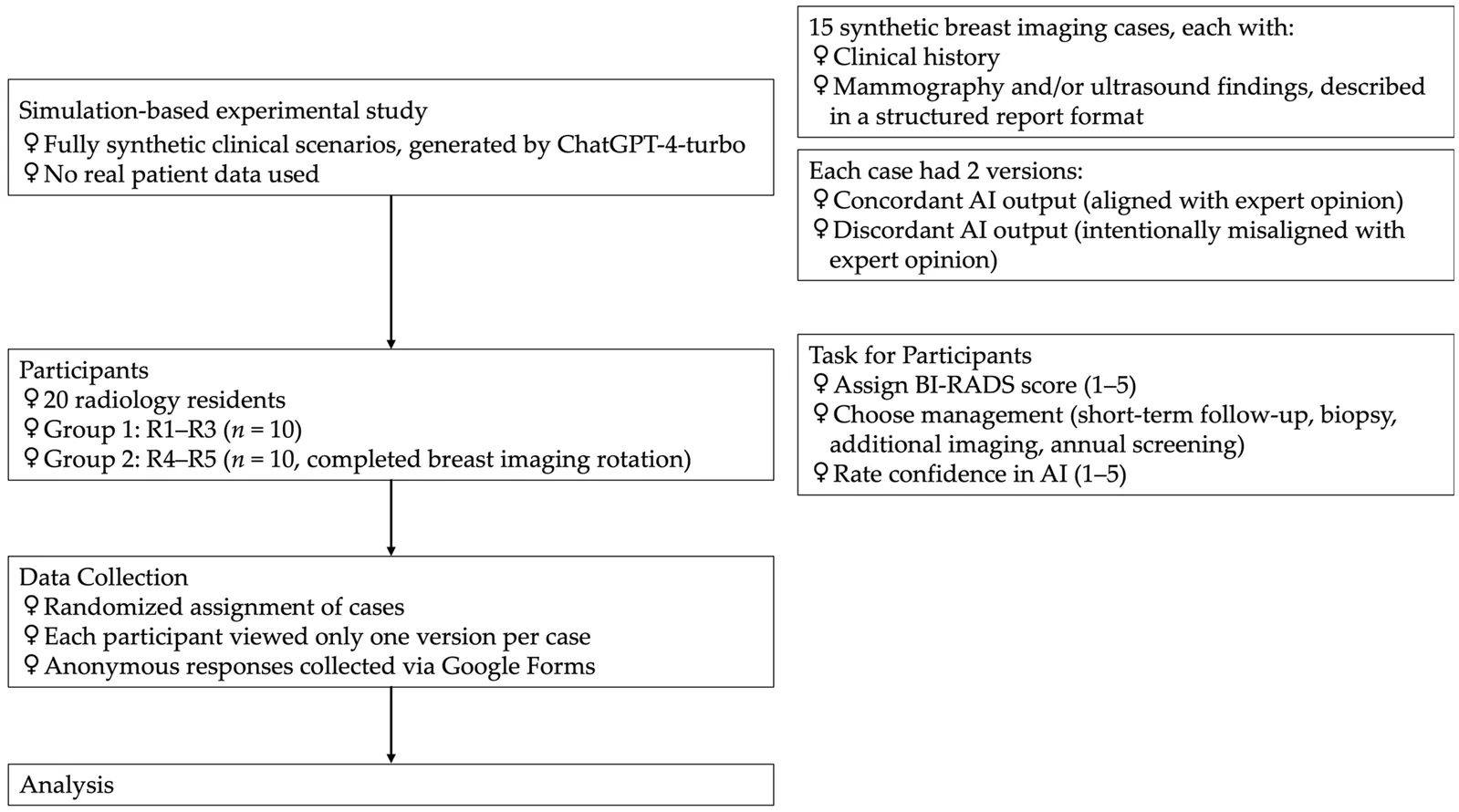
Flowchart of the study design. AI: artificial intelligence; BI-RADS: Breast Imaging Reporting and Data System; R: year of residency.

A total of 20 residents participated in the study: 10 in Group 1 and 10 in Group 2 (Figure 1).

**Figure 2:**
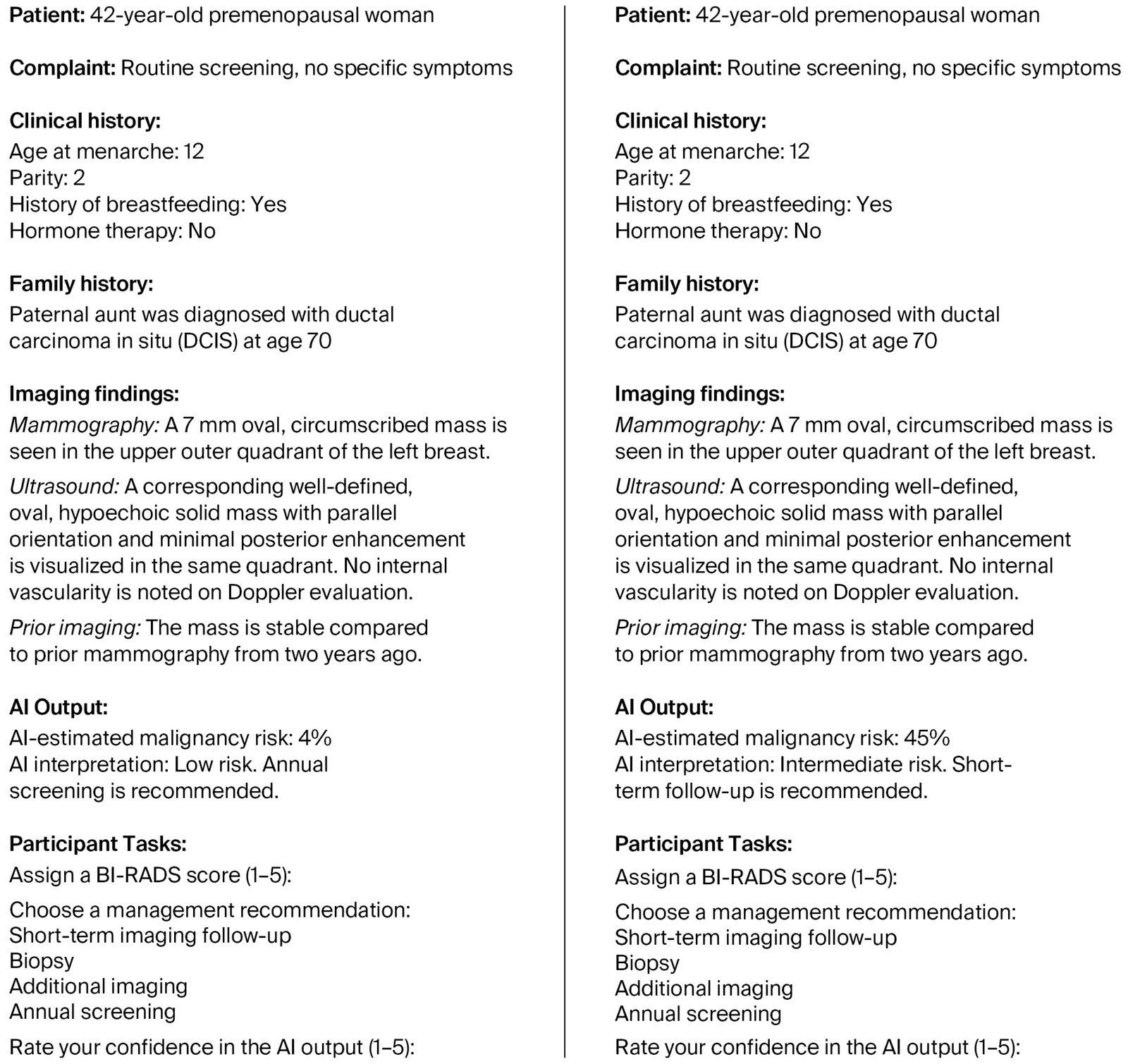
Representative benign case template generated by ChatGPT-4 Turbo. The left-hand panel presents the concordant version, where the LLM correctly identifies a low risk of malignancy, while the right-hand panel shows the discordant version, where the LLM provides an intentionally erroneous intermediate risk estimate to assess automation bias. AI: artificial intelligence; BI-RADS: Breast Imaging Reporting and Data System; DCIS: ductal carcinoma in situ.

Cases were presented as structured text-based clinical vignettes incorporating simulated mammographic and/or ultrasonographic findings in descriptive form, rather than actual DICOM images. This study therefore evaluated resident decision-making in response to reported imaging findings and AI-generated risk estimates—not direct radiological image interpretation. All synthetic clinical scenarios were generated using ChatGPT-4 Turbo (OpenAI, 2024). Case parameters (patient age, menopausal status, and diagnostic category: benign, intermediate/gray zone, or malignant) and prompts were jointly planned and specified by two radiologists with 23 and 4 years of experience in breast radiology, respectively. Typical prompts included instructions such as “Create a plausible, benign case for a 42-year-old premenopausal woman. Include clinical details [relevant symptoms, clinical history (age at menarche, parity, history of breastfeeding, hormone therapy), and family history] and structured US and/or mammography findings consistent with a benign diagnosis.” Based on these parameters, the model generated the entire case in a structured report format (Figure 2). Before inclusion in the survey, the two radiologists jointly reviewed every case by consensus, with editing when necessary to ensure clinical coherence, plausibility, adherence to imaging standards, and internal consistency between the described imaging findings and the assigned diagnostic category; no case was included without this validation step. The same consensus review established the ground-truth diagnostic category and reference management recommendation for each case. Because synthetic cases have no pathological confirmation or clinical follow-up, this expert consensus served as the reference standard for scoring resident accuracy, rather than an external pathological or outcome-based standard. Model outputs were standardized by removing non-prose formatting and ancillary segments in order to maintain comparable readability assessments, consistent with prior studies [19,20,21]. The normalized text was retained as plain prose with standardized punctuation; no lexical simplification was applied. We archived GPT-4 responses for every case under each prompt to ensure reproducibility. No real patient data or medical images were used; all content was entirely fictional. A total of 15 synthetic cases were generated, comprising 5 benign, 5 intermediate, and 5 malignant cases. Each case had two versions:

- Concordant AI version: An AI-generated risk estimate that aligned with expert opinion was included.
- Discordant AI version: An AI-generated risk estimate that intentionally misaligned with expert opinion was included. All discordant estimates fell in a different risk band from the one corresponding to the case’s ground-truth category, with a correspondingly different AI-generated interpretation statement, and differed from the concordant estimates by 33 to 80 percentage points (median: 41) (Supplementary Table S1).

In all cases, the participants were shown the following standardized statement: “AI estimated malignancy risk: XX%”. In order to ensure a balanced distribution across risk levels and to facilitate subgroup comparisons, AI-estimated malignancy risk scores were stratified into three categories: low (0–32%), intermediate (33–66%), and high (67–100%). The LLM generated the numeric risk percentage for each case as part of the structured case output, in line with the case’s intended risk category. When a generated value was judged to be inconsistent with that category or clinically implausible, the two radiologists adjusted it during the consensus review described above.

The case scenarios and response forms were presented through Google Forms, and all responses were submitted anonymously. To prevent bias, we employed a randomized, counterbalanced between-subjects design: two survey versions were created, each containing the opposite AI-concordance version of every case, and participants were randomly allocated to one version, ensuring that no participant saw the same case twice. Participants in each group were randomly assigned to a form, with a randomized case order to mitigate learning or fatigue effects. To verify accurate grouping, residents were asked a single question regarding their breast imaging rotation status. Self-reported responses were then cross-checked against institutional training records, which confirmed full concordance for all participants. Participants were instructed to base their responses independently on the information provided in each case scenario and to complete the following tasks:

1. Assign a BI-RADS score (1–5).
2. Select a management recommendation:
  - Short-term imaging follow-up;
  - Biopsy;
  - Additional imaging;
  - Annual screening (i.e., no follow-up required).
3. Rate your confidence in the AI risk estimate (1 = not at all confident, 5 = fully confident).

BI-RADS scores were assigned using the standard 1–5 scale without sub-categorization (e.g., 4A/4B/4C). For accuracy scoring, categories were grouped according to their clinical management implications: BI-RADS 1–2 (benign), BI-RADS 3 (intermediate/gray zone), and BI-RADS 4–5 (malignant). A resident’s response was considered to be accurate if it fell within the same group as the ground-truth category. BI-RADS accuracy and management accuracy were scored independently: a resident’s management recommendation was evaluated against the reference management plan regardless of the accuracy of their concurrent BI-RADS assignment, and vice versa. A response was classified as demonstrating automation bias if, in the discordant AI condition, the resident’s assigned BI-RADS category or selected management recommendation matched the AI’s incorrect suggestion—provided as an explicit “AI interpretation” statement alongside the numeric risk estimate—rather than the correct ground-truth category or reference management plan. Responses in which the resident’s answer diverged from the AI’s suggestion—i.e., matched the ground truth despite discordant AI input—were classified as not demonstrating automation bias for that trial.

### Statistical Analysis

Statistical analyses were performed using SPSS version 20.0 (IBM Corp., Armonk, NY, USA). The Shapiro–Wilk test was used to assess data normality. Continuous and ordinal variables were presented as the mean ± standard deviation (SD) or median [interquartile range (IQR)], while categorical variables were expressed as frequencies and percentages. A *p*-value < 0.05 was considered statistically significant. The Wilcoxon signed-rank test and the McNemar–Bowker test were used to compare BI-RADS scores and management shifts, respectively. Diagnostic accuracy rates were calculated and compared between resident groups using the chi-squared test or Fisher’s exact test, as appropriate. The impact of AI concordance on accuracy was first assessed using the McNemar test within each group. Confidence in AI risk estimates was compared between training levels using the Mann–Whitney U test and between AI versions using the Wilcoxon signed-rank test. Subsequently, to evaluate independent predictors of AB while accounting for reader-level clustering inherent to the repeated-measures design (15 cases per reader, yielding 300 evaluations across 20 residents), multivariable Generalized Estimating Equations (GEE) binary logistic regression models were constructed; AB—defined as adopting an incorrect decision under discordant AI guidance—served as the binary dependent variable. Individual reader ID (*n* = 20 clusters) was designated as the clustering unit. An exchangeable working correlation matrix was selected, assuming uniform intra-reader correlation across randomly sequenced cases, and confirmed by the quasi-likelihood under the independence model criterion (QIC). Robust (Huber–White/sandwich) standard errors were used to ensure valid parameter inference. Model stability was verified with an adequate event-per-variable ratio, and small-sample bias-corrected sandwich variance estimation (Mancl–DeRouen correction) was evaluated to assess parameter stability across the 20 clusters. The results are reported as adjusted odds ratios (ORs) with 95% confidence intervals (CIs) and Wald test *p*-values.

## 3. Results

A total of 300 evaluations were performed across 15 synthetic cases from 20 radiology residents. The distribution of the participants’ BI-RADS assignments and management recommendations, stratified by AI concordance, is presented in Table 1.

The diagnostic accuracy for management recommendations was significantly higher in concordant cases compared to discordant cases (84.0% vs. 67.3%, respectively; *p* = 0.008). While a similar trend was observed in BI-RADS assignment accuracy (72.7% vs. 62.0%), the difference did not reach statistical significance (*p* = 0.060).

Residents’ confidence in AI risk estimates varied substantially based on the concordance of the AI output. The median confidence score was significantly higher for concordant versions (median: 4.0, IQR: 4.0–5.0) than for discordant versions (median: 2.0, IQR: 1.0–3.0; *p* < 0.001).

### 3.1. Training Level and Diagnostic Performance

**Figure 3:**
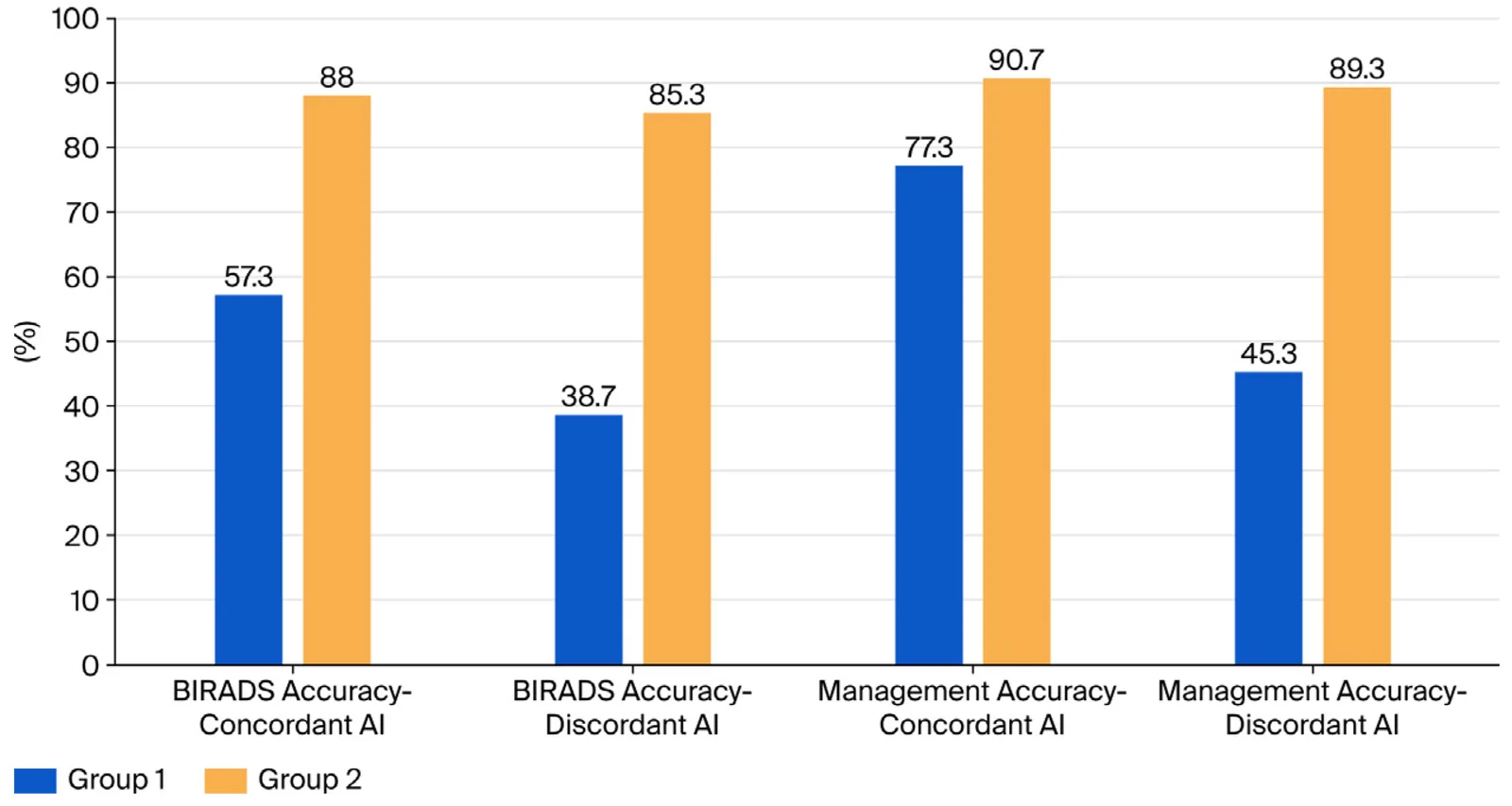
Diagnostic accuracy of residents, stratified by training level and AI concordance. Note the significant decline in diagnostic performance for early-stage residents (light) when faced with discordant AI recommendations, contrasted with the stable performance of advanced residents (dark). Values are presented as percentages. AI: artificial intelligence; BI-RADS: Breast Imaging Reporting and Data System.

The diagnostic accuracy of the residents was assessed by BI-RADS assignments and management recommendations (Table 2 and Figure 3). Advanced residents achieved significantly higher accuracy than early-stage residents across all assessment categories (*p* < 0.001). For advanced residents, both BI-RADS and management accuracy remained stable and high, showing no statistically significant decline when compared with discordant AI (BI-RADS: 88.0% to 85.3%, *p* = 0.695; management: 90.7% to 89.3%, *p* = 0.733). In contrast, early-stage residents were significantly impacted by discordant AI outputs. Their BI-RADS assignment accuracy dropped from 57.3% to 38.7% (*p* = 0.049), while their clinical management accuracy declined even more, from 77.3% to 45.3% (*p* = 0.006).

### 3.2. Subgroup Analysis by Case Category

The influence of AI concordance was further analyzed across case categories (Table 3 and Table 4). In malignant cases, discordant AI severely affected early-stage residents, whose BI-RADS and management accuracy dropped to 16.0% and 48.0%, respectively (*p* < 0.05). Conversely, advanced residents maintained high BI-RADS and management accuracy regardless of AI concordance (84.0% vs. 76.0% and 88.0% vs. 96.0%). For intermediate cases, early-stage residents’ accuracy under discordant AI declined on both metrics (BI-RADS: 68.0% to 52.0%; management: 64.0% to 40.0%), although neither reached statistical significance; advanced residents remained more stable (BI-RADS: 92.0% to 88.0%; management: 88.0% to 80.0%). In benign cases, AB persisted among early-stage residents, with management accuracy decreasing from 76.0% to 48.0% and BI-RADS accuracy decreasing from 60.0% to 48.0% due to incorrect high-risk AI prompts. Advanced residents showed maximum stability, maintaining 92.0% management accuracy and a slight, non-significant increase in BI-RADS accuracy (88.0% to 92.0%), despite discordant outputs.

### 3.3. AI Confidence Scores

Both resident groups exhibited high levels of confidence when the AI provided concordant results. However, advanced residents demonstrated significantly greater confidence in these correct estimates than early-stage residents (median: 5.0 [IQR: 4.0–5.0] vs. 4.0 [IQR: 3.0–5.0], *p* < 0.001). Conversely, when faced with discordant AI versions, advanced residents showed a sharp decline in confidence, while early-stage residents maintained a moderate level of trust (median: 1.0 [IQR: 1.0–2.0] vs. 3.0 [IQR: 2.0–3.0], *p* < 0.001).

### 3.4. Predictors of AB

The multivariable GEE binary logistic regression analysis—accounting for clustering across the 20 readers (300 evaluations)—included training level (early-stage vs. advanced) and case category (benign, intermediate, and malignant) as independent variables, with malignant cases serving as the reference category. Our results demonstrated that training level was the only significant independent predictor of AB (Table 5). Advanced residents were significantly less likely to align with incorrect AI recommendations compared with early-stage residents (OR: 0.09, 95% CI: 0.03–0.30, *p* < 0.001). In contrast, no statistically significant differences in the likelihood of AB were observed between malignant cases and other categories (*p* > 0.05).

## 4. Discussion

This study underscores that susceptibility to AB in breast imaging is significantly influenced by the resident’s level of clinical experience. The primary finding reveals a marked disparity in susceptibility to AB between early-stage and advanced radiology residents. Advanced residents who had completed a dedicated breast imaging rotation exhibited substantially greater resilience to erroneous AI outputs. This protective effect was most pronounced in malignant cases, where misleading AI outputs nearly halved the diagnostic accuracy among early-stage residents, whereas advanced residents maintained consistent performance regardless of AI concordance. Notably, even under concordant AI guidance, BI-RADS accuracy among early-stage residents in malignant cases was only 44.0%—considerably lower than their management accuracy (92.0%) in the same condition. This suggests that early-stage residents’ difficulty with malignant BI-RADS categorization may partially reflect an inherent skill gap independent of AI influence, rather than being attributable solely to discordant AI exposure. Interestingly, advanced residents’ accuracy demonstrated a modest increase under discordant AI in two subgroups: BI-RADS accuracy for benign cases (88.0% to 92.0%), and management accuracy for malignant cases (88.0% to 96.0%). Neither of these cases was statistically significant; therefore, this pattern may simply reflect random variation rather than a true effect. This situation could be speculatively interpreted as follows: experienced residents who encountered AI outputs contradicting their own impressions may have re-evaluated the case with heightened vigilance and greater care—a response plausibly triggered by the sharp drop in their confidence under discordant conditions (median confidence 5.0 to 1.0, *p* < 0.001). This meticulous evaluation could, in some instances, have outperformed the more routine engagement seen when AI and clinical impression agreed. This interpretation remains hypothesis-generating and would need confirmation in a larger, adequately powered sample. It should also be acknowledged that experience is not an unconditional safeguard: overconfidence and confirmation bias have been documented even among senior clinicians, who may dismiss a correct AI alert because it conflicts with an already-formed impression [22]. Our design, which measured accuracy rather than the reasoning process behind each decision, cannot distinguish genuine critical re-evaluation from confidence-driven dismissal of the AI’s input. Furthermore, the data show that AB is more evident in clinical management recommendations than in BI-RADS assignments. These findings suggest that even when trainees accurately identify lesion characteristics, their ultimate clinical decisions remain vulnerable to misleading algorithmic input.

Our findings reveal the inverse relationship between clinical experience and susceptibility to AB. While advanced residents maintained stable accuracy regardless of AI concordance, early-stage residents showed a dramatic decline in management accuracy when the AI output was discordant. This suggests that as residents progress in their training, they develop a more refined clinical judgment that allows them to override incorrect AI recommendations. This aligns with the “dual-process theory” of clinical reasoning [23,24,25], in which experienced clinicians rely more on intuitive, pattern-recognition-based Type 2 thinking, whereas less experienced clinicians may rely more on external aids such as AI, making them more vulnerable to systemic errors. Particularly concerning is the performance of early-stage residents in cases involving malignancy. When AI incorrectly suggested a low risk for a malignant lesion, management accuracy dropped to 48%. This finding has critical implications for patient safety in real-world clinical settings: if junior residents or non-expert readers use AI as an alternative to their own decision-making, the risk of missing cancers increases significantly. Our findings are consistent with the previous literature. In a prospective study with 27 radiologists investigating how AI outputs can affect diagnostic accuracy at three levels of experience when reading mammograms, Dratsch et al. demonstrated that when AI suggests an incorrect BI-RADS category, diagnostic accuracy collapses across all experience levels, but inexperienced radiologists are the most biased [15]. In a closely related study, ten radiology residents with varying levels of training in breast imaging were asked to evaluate mammograms—first independently, and subsequently with the aid of AI. It was found that AI altered the final BI-RADS category in only 9.7% of the readings but influenced the level of diagnostic confidence in 19.2%, with confidence increases substantially outnumbering the decreases. Notably, when confidence changed without an accompanying BI-RADS modification, trust in the AI system—rather than a re-examined finding—was the reported reason in 83.4% of instances, closely paralleling the pattern of AI-driven confidence shifts observed in our own resident cohort [26]. In another study on AI-assisted cerebral aneurysm detection, false-positive AI marks increased suspicion among all readers. However, inexperienced radiologists were strongly influenced, whereas highly experienced neuroradiologists demonstrated greater resilience and made fewer AI-driven overcalls [27].

This study demonstrates a significant discrepancy between AI’s influence on BI-RADS scoring and on final clinical management recommendations. While the overall decline in BI-RADS accuracy under discordant AI conditions showed a non-significant trend, clinical management accuracy was significantly impaired across readers. Because the two outcomes were scored independently, this divergence cannot be attributed to a scoring dependency between them—a resident could assign a correct BI-RADS category while still selecting an incorrect management plan, or vice versa. Therefore, the discrepancy reflects two genuinely distinct components of clinical decision-making. To statistically support this observation, we analyzed the paired concordance between BI-RADS categorization and management selection under discordant AI exposure: among cases where residents accurately identified the true BI-RADS category, 19.4% nonetheless altered their clinical management to follow the erroneous AI estimate. Conversely, assigning an incorrect BI-RADS score while retaining the correct management occurred in only 4.3% of evaluations, demonstrating a statistically significant task decoupling (*p* = 0.003). This empirical disparity suggests that trainees often retain cognitive independence during standardized feature recognition and categorical scoring, yet they surrender decision autonomy when formulating actionable, downstream patient interventions. This behavior can be described as a delegation of responsibility, wherein trainees rely on the AI’s risk estimate for psychological reassurance. Furthermore, the significant reduction in confidence scores during discordant cases indicates that, although residents can recognize a conflict between their assessments and the AI output, this awareness does not always prevent changes in their clinical recommendations. Moreover, this discrepancy highlights a failure in trust calibration: advanced residents reduced their trust after identifying an error, whereas in situations of uncertainty, early-stage residents may place greater trust in the algorithm’s numerical certainty than in their own visual intuition. The pressure to avoid missing malignant cases may also prompt less experienced residents to follow a high-risk AI estimate, such as a biopsy recommendation, even when imaging features appear benign. Consistent with this perspective, previous research demonstrates that such errors of commission often result both from overestimating the system’s capabilities and from a lack of confidence in individual diagnostic skills [13,15,28].

AB is a well-documented cognitive error where clinicians over-rely on automated systems, leading to a failure in independent critical thinking [13,29,30,31]. In radiology, this manifests as two error types: commission, acting on incorrect AI prompts to perform unnecessary interventions; and omission, failing to detect abnormalities when the system fails to flag them [15,28,29,32]. Previous studies have specified several factors influencing AB in radiology: First, experience is paramount; while all radiologists are susceptible, less experienced readers are significantly more likely to accept erroneous AI suggestions [15]. Even expert radiologists may be affected by such biases when working under high cognitive load [15,22]. Second, case types and cancer prevalence in the clinical environment alter diagnostic thresholds. In high-prevalence settings, AI support amplifies the shift toward higher sensitivity, further aligning clinician decisions with algorithmic operating points [33]. Finally, task difficulty and the clarity of malignant signs are critical [33]. For instance, when image signs are clear, radiologists are more likely to notice (and correct) mistakes made by algorithms. In contrast, in more subtle cases, incorrect AI results can have a greater impact. In this study, training level emerged as the only statistically significant independent predictor of AB, whereas case category showed no significant association. However, training level should not be interpreted as a universal safeguard against AB. Automation bias is a multifactorial phenomenon: factors outside the scope of our design—biases embedded in the AI model’s training data, overconfidence effects among experienced clinicians, the opacity of its decision logic, and the adequacy of institutional oversight protocols—likely act independently of a clinician’s experience level, contributing to AB susceptibility on their own terms. Given the wide confidence intervals for case category (e.g., intermediate vs. malignant: OR 2.02, 95% CI 0.81–5.04), this study may have been underpowered to detect a true effect of lesion difficulty, and the absence of a significant association should not be interpreted as evidence that diagnostic difficulty plays no role in AB susceptibility. This null finding may partially reflect the standardized nature of our experimental design, which reduced external factors known to amplify case-difficulty effects in real-world settings, such as high cognitive load and time pressure; however, we cannot rule out the possibility that it also reflects limited statistical power given the sample size. Distributing cases evenly in a 1:1:1 ratio was intended to reduce the influence of the prevalence effect commonly observed in real clinical settings, although this was not directly measured in our design.

The key strength of our study is its experimental framework. We used consensus-reviewed synthetic clinical scenarios and a randomized, counterbalanced between-subjects design to isolate the specific impact of AB at two different levels of radiological training. By enriching these cases with detailed demographic data and clinical histories, we aimed to simulate the cognitive complexity of real-world breast cancer risk assessments. This study underscores the potential for LLMs to transform the methodology of medical research. By creating realistic clinical scenarios, LLMs enable large, affordable simulation studies that were previously impossible, allowing institutions to test how clinicians and AI interact in a safe setting before using the technology in real cases. However, several limitations must be acknowledged: First, the small sample size may limit the generalizability of our findings. While our clustered GEE model demonstrated robust convergence and stability under small-sample bias corrections across 20 resident clusters, the marked protective effect observed for training level (OR: 0.09) should be validated in larger multi-institutional cohorts with broader clinical exposure. Second, this study evaluated decision-making in response to structured, text-based clinical vignettes incorporating simulated mammographic and/or ultrasonographic findings, rather than actual DICOM images or direct radiological image interpretation. Consequently, our findings should not be generalized to AI tools that provide direct image-based outputs (e.g., visual heatmaps or pixel-level saliency maps), and the extent to which text-based performance reflects real-world image-based diagnostic behavior remains uncertain. Third, as the AI risk estimates were simulated to maintain experimental control, the results may not replicate the unpredictable dynamics or hallucinations of live models in routine workflows. Additionally, as responses were collected anonymously via an unproctored online form, we cannot fully exclude the possibility of external consultation or extended deliberation time during case evaluation, which may not reflect the time-constrained nature of real-world clinical decision-making.

The integration of AI into breast imaging has shifted from a question of feasibility to one of implementation. As more AI-enabled medical devices receive approval, educators must ensure that these tools are used safely and effectively. Our research adds to the discussion of the unintended effects of AI—particularly the risk of over-reliance by future radiologists and the loss of independent clinical judgment. We recommend updating training programs to include strategies that encourage critical thinking and gradually increase independence during residency [30]. Providing training on AB can also help clinicians continue to verify results on their own [11,12,34]. Research indicates that presenting the confidence level of AI systems encourages clinicians to critically evaluate AI-generated results. Therefore, the use of explainable models—such as saliency maps, visual explanations, and explicit rationales—is essential, as they allow clinicians to observe and understand the decision-making processes of AI systems [15,35,36,37,38,39,40]. Beyond training level, the opacity of AI decision-making is itself a structural driver of AB. When clinicians cannot inspect the basis of a risk estimate, trust calibration suffers regardless of experience—a pattern often described as the “black-box effect” [22]. Explainability tools alone do not reliably resolve this: AI-generated explanations can paradoxically reinforce unwarranted trust rather than prompting critical engagement [22]. Institutional oversight mechanisms—e.g., multidisciplinary review of discordant AI–clinician cases, periodic audit of model outputs against ground truth [38]—may therefore complement individual-level explainability rather than substituting for it. Active learning strategies that continuously retrain models on diverse, balanced datasets could also reduce the training-data biases that predispose AI systems to systematic errors in specific case categories [38], although our study was not designed to evaluate this mechanism. Several studies recommend that clinicians document their initial decisions before reviewing AI outputs and provide justification when overriding AI recommendations; these practices may help maintain clinical focus and reduce the risk of uncritical acceptance of AI advice [12,34]. Finally, protocol validation is necessary to ensure that AI tools are effective across diverse populations and to maintain long-term clinical safety.

In conclusion, LLM-generated risk estimates can bring a real risk of AB, especially for early-stage trainees. Clinical experience is important for mitigating machine errors among residents, underscoring the need for robust subspecialty training. However, clinical experience is only one of several factors that shape resilience to automation bias, and our findings should not be read as evidence that training level alone is sufficient to prevent it—model transparency, institutional oversight, and dataset quality matter just as much to a safe deployment strategy. AI should be seen as a tool that supports, not replaces, human expertise. To reduce AB in clinical decision-making, AI tools need to be integrated into human-centered, reliable workflows that combine clinician training with system-level safeguards.

## Figures and Tables

**Table 1:** Distribution of Radiologic Assessments by AI Version.

Assessment	Concordant AI (*n* = 150)	Discordant AI (*n* = 150)	Δ (95% CI)	Effect Size	*p-*Value
BI-RADS Accuracy	109 (72.7%)	93 (62.0%)	10.7% (−0.2–21.6)	OR: 1.63	0.060
Management Accuracy	126 (84.0%)	101 (67.3%)	16.7% (6.9–26.5)	OR: 2.56	0.008 *
AI Confidence Score	4 [4,5]	2 [1,2,3]	2.0 (1.0–2.0)	*r* = 0.74	<0.001 *

Values are expressed as *n* (%) or mean ± SD or median [IQR]. Δ represents the absolute percentage difference with 95% CI for accuracies, and the median difference for the confidence score. Effect size is reported as OR for categorical diagnostic accuracy and as Wilcoxon effect size (*r*) for the ordinal confidence score; * Significance at *p* < 0.05; AI: artificial intelligence; BI-RADS: Breast Imaging Reporting and Data System; CI: confidence interval; IQR: interquartile range; OR: odds ratio; SD: standard deviation.

**Table 2:** Diagnostic Performance by Training Level and AI Concordance.

Assessment Metric	Group	Concordant AI (*n* = 75)	Discordant AI (*n* = 75)	Δ (95% CI)	Effect Size	*p*-Value
BI-RADS Accuracy	Group 1	43 (57.3%)	29 (38.7%)	18.7% (3.2–34.1)	2.13	0.049 *
	Group 2	66 (88.0%)	64 (85.3%)	2.7% (−8.3–13.7)	1.26	0.695
	Δ (95% CI)	30.7% (16.9–44.5)	46.7% (32.7–60.6)			
	Effect Size	5.46	9.24			
	*p*-value	<0.001 *	<0.001 *			
Management Accuracy	Group 1	58 (77.3%)	34 (45.3%)	32.0% (17.3–46.7)	4.12	0.006 *
	Group 2	68 (90.7%)	67 (89.3%)	1.3% (−8.5–11.2)	1.16	0.733
	Δ (95% CI)	13.3% (1.5–25.2)	44.0% (30.2–57.8)			
	Effect Size	2.85	10.11			
	*p*-value	0.045 *	<0.001 *			

Values are presented as *n* (%). Δ indicates the absolute percentage difference with 95% CIs. Effect size is expressed as the OR; * Significance at *p* < 0.05; AI: artificial intelligence; BI-RADS: Breast Imaging Reporting and Data System; CI: confidence interval; OR: odds ratio.

**Table 3:** BI-RADS Accuracy Stratified by Case Category and AI Concordance.

Case Category	Group	Concordant AI BI-RADS Accuracy (*n* = 25)	Discordant AI BI-RADS Accuracy (*n* = 25)	Δ (95% CI)	Effect Size	*p*-Value
Benign	Group 1	15 (60.0%)	12 (48.0%)	12.0% (−14.8–38.8)	1.63	0.343
	Group 2	22 (88.0%)	23 (92.0%)	−4.0% (−21.5–13.5)	0.64	0.589
Intermediate	Group 1	17 (68.0%)	13 (52.0%)	16.0% (−10.4–42.4)	1.96	0.066
	Group 2	23 (92.0%)	22 (88.0%)	4.0% (−13.5–21.5)	1.57	0.640
Malignant	Group 1	11 (44.0%)	4 (16.0%)	28.0% (4.1–51.9)	4.13	0.017 *
	Group 2	21 (84.0%)	19 (76.0%)	8.0% (−13.9–29.9)	1.66	0.642

Values are presented as *n* (%). Δ indicates the absolute percentage difference with 95% CIs. Effect size is expressed as the OR; * Significance at *p* < 0.05; AI: artificial intelligence, BI-RADS: Breast Imaging Reporting and Data System, CI: confidence interval; OR: odds ratio.

**Table 4:** Management Accuracy Stratified by Case Category and AI Concordance.

Case Category	Group	Concordant AI Management Accuracy (*n* = 25)	Discordant AI Management Accuracy (*n* = 25)	Δ (95% CI)	Effect Size	*p*-Value
Benign	Group 1	19 (76.0%)	12 (48.0%)	28.0% (1.7–54.3)	3.43	0.131
	Group 2	24 (96.0%)	23 (92.0%)	4.0% (−9.7–17.7)	2.09	0.564
Intermediate	Group 1	16 (64.0%)	10 (40.0%)	24.0% (−3.1–51.1)	2.67	0.460
	Group 2	22 (88.0%)	20 (80.0%)	8.0% (−12.4–28.4)	1.83	0.317
Malignant	Group 1	23 (92.0%)	12 (48.0%)	44.0% (20.6–67.4)	12.46	<0.001 *
	Group 2	22 (88.0%)	24 (96.0%)	−8.0% (−23.8–7.8)	0.30	0.157

Values are presented as *n* (%). Δ indicates the absolute percentage difference with 95% CIs. Effect size is expressed as the OR; * Significance at *p* < 0.05; AI: artificial intelligence; CI: confidence interval; OR: odds ratio.

**Table 5:** Logistic Regression Analysis for Predictors of Automation Bias.

Predictor	OR	95% CI	*p*-Value
Training Level (Advanced vs. Early)	0.09	0.03–0.30	<0.001 *
Case Category			
Benign (vs. Malignant)	1.13	0.44–2.91	0.796
Intermediate (vs. Malignant)	2.02	0.81–5.04	0.132

Values were estimated using multivariable GEE binary logistic regression with individual residents specified as the clustering unit (*K* = 20 clusters, *N* = 300 observations), an exchangeable correlation structure, and robust standard errors. Model stability was confirmed using small-sample bias-corrected variance estimation (Mancl–DeRouen correction); CI: Confidence Interval; GEE: Generalized Estimating Equations; OR: Odds Ratio; * Significance at *p* < 0.05.

## Data Availability

The data presented in this study are available from the corresponding author upon reasonable request. The data are not publicly available due to participant privacy and ethical restrictions.

## Supplementary Materials

The following supporting information can be downloaded at https://www.mdpi.com/article/10.3390/diagnostics16193241/s1: Table S1: AI-estimated malignancy risk percentages presented to participants for the concordant and discordant versions of each of the 15 synthetic cases.

## Abbreviations

The following abbreviations are used in this manuscript:

AI	Artificial Intelligence
LLM	Large Language Model
Chat-GPT4	Generative Pre-Trained Transformer 4
BI-RADS	Breast Imaging Reporting and Data System
AB	Automation Bias
GEE	Generalized Estimating Equations
US	Ultrasound
MRI	Magnetic Resonance Imaging
R	Year of Residency
DCIS	Ductal Carcinoma In Situ
SD	Standard Deviation
IQR	Interquartile Range
CI	Confidence Interval
OR	Odds Ratio
