# Supplementary material for: The Influence of Large Language Model-Generated Artificial Intelligence Risk Estimates on Clinical Decision-Making in Breast Imaging: A Simulation-Based Study
Source: Diagnostics (Basel). 2026 Oct 7;16(19):3241. doi: 10.3390/diagnostics16193241 (PMC13664299; doi:10.3390/diagnostics16193241)
Supplement: Supplementary file 1 [file diagnostics-16-03241-s001.zip › diagnostics-4588501-supplementary.pdf]

Table S1. AI-estimated malignancy risk percentages presented to participants for the concordant and discordant versions of each of the 15 synthetic cases.

Case No.	Case Category	Concordant AI Estimate (%)	Discordant AI Estimate (%)
1	Benign	4	45
2	Benign	2	69
3	Benign	4	58
4	Benign	5	85
5	Benign	8	49
6	Intermediate	34	78
7	Intermediate	40	5
8	Intermediate	48	10
9	Intermediate	49	9
10	Intermediate	47	80
11	Malignant	93	37
12	Malignant	85	5
13	Malignant	89	35
14	Malignant	91	51
15	Malignant	95	29

Note: AI-estimated malignancy risk scores were stratified into three categories—low (0-32%), intermediate (33-66%), and high (67-100%)—as described in the Methods. For each case, the concordant AI estimate falls within the band consistent with the case's ground-truth category (benign: low; intermediate: intermediate; malignant: high), while the discordant AI estimate was intentionally shifted to a different band to create a misaligned AI suggestion.
